# Treatment of periodontal intrabony defects using autologous periodontal ligament stem cells: a randomized clinical trial

**DOI:** 10.1186/s13287-016-0288-1

**Published:** 2016-02-19

**Authors:** Fa-Ming Chen, Li-Na Gao, Bei-Min Tian, Xi-Yu Zhang, Yong-Jie Zhang, Guang-Ying Dong, Hong Lu, Qing Chu, Jie Xu, Yang Yu, Rui-Xin Wu, Yuan Yin, Songtao Shi, Yan Jin

**Affiliations:** State Key Laboratory of Military Stomatology, Department of Periodontology, School of Stomatology, Fourth Military Medical University, Xi’an, Shannxi P. R. China; State Key Laboratory of Military Stomatology, Research and Development Center for Tissue Engineering, School of Stomatology, Fourth Military Medical University, Xi’an, Shannxi P. R. China; Department of Anatomy and Cell Biology, School of Dental Medicine, University of Pennsylvania, 240 South 40th Street, Philadelphia, PA 19104 USA

**Keywords:** Stem cell-therapy, Periodontitis, Periodontal regeneration, Cell sheet, Tissue engineering, Translational medicine

## Abstract

**Background:**

Periodontitis, which progressively destroys tooth-supporting structures, is one of the most widespread infectious diseases and the leading cause of tooth loss in adults. Evidence from preclinical trials and small-scale pilot clinical studies indicates that stem cells derived from periodontal ligament tissues are a promising therapy for the regeneration of lost/damaged periodontal tissue. This study assessed the safety and feasibility of using autologous periodontal ligament stem cells (PDLSCs) as an adjuvant to grafting materials in guided tissue regeneration (GTR) to treat periodontal intrabony defects. Our data provide primary clinical evidence for the efficacy of cell transplantation in regenerative dentistry.

**Methods:**

We conducted a single-center, randomized trial that used autologous PDLSCs in combination with bovine-derived bone mineral materials to treat periodontal intrabony defects. Enrolled patients were randomly assigned to either the Cell group (treatment with GTR and PDLSC sheets in combination with Bio-oss^®^) or the Control group (treatment with GTR and Bio-oss^®^ without stem cells). During a 12-month follow-up study, we evaluated the frequency and extent of adverse events. For the assessment of treatment efficacy, the primary outcome was based on the magnitude of alveolar bone regeneration following the surgical procedure.

**Results:**

A total of 30 periodontitis patients aged 18 to 65 years (48 testing teeth with periodontal intrabony defects) who satisfied our inclusion and exclusion criteria were enrolled in the study and randomly assigned to the Cell group or the Control group. A total of 21 teeth were treated in the Control group and 20 teeth were treated in the Cell group. All patients received surgery and a clinical evaluation. No clinical safety problems that could be attributed to the investigational PDLSCs were identified. Each group showed a significant increase in the alveolar bone height (decrease in the bone-defect depth) over time (*p* < 0.001). However, no statistically significant differences were detected between the Cell group and the Control group (*p* > 0.05).

**Conclusions:**

This study demonstrates that using autologous PDLSCs to treat periodontal intrabony defects is safe and does not produce significant adverse effects. The efficacy of cell-based periodontal therapy requires further validation by multicenter, randomized controlled studies with an increased sample size.

**Trial Registration:**

NCT01357785 Date registered: 18 May 2011.

**Electronic supplementary material:**

The online version of this article (doi:10.1186/s13287-016-0288-1) contains supplementary material, which is available to authorized users.

## Background

Periodontitis is an inflammatory disease that causes pathological alterations in tooth-supporting tissues, which can lead to tooth loss if left untreated. National surveys have shown that the majority of adults suffer from moderate periodontitis, and up to 15 % of the population is affected by severe generalized periodontitis at some stage of their lives [1, 2]. The significant burden of periodontal disease and its impact on general health and patient quality of life suggest a clinical need for the effective management of this condition [3–5]. The ultimate goal of periodontal therapy is the predictable regeneration of the functional attachment apparatus that is destroyed by periodontitis, which involves at least three unique tissues, including the cementum, periodontal ligament (PDL), and alveolar bone. To date, several regenerative procedures have been developed in an attempt to treat periodontitis, including guided tissue regeneration (GTR), bone graft placement, and the use of bioactive agents, such as growth factors (reviewed in [5–8]). However, the current therapeutic techniques used either alone or in combination have limitations in producing complete and predicable regeneration, especially in advanced periodontal defects. In these cases, remaining deep intraosseous defects following periodontal therapy are high-risk sites for the further progression of periodontitis (reviewed in [9–11]).

According to histological evidence, the GTR technique combined with grafting materials, such as Bio-oss^®^ (Geistlich Pharm. AG, Volhusen, Switzerland) and autologous bone, is partially effective at treating periodontal defects; however, the currently available GTR-based therapies remain rudimentary and show poor clinical predictability (reviewed in [7, 8, 11]). Recent advances in stem cell biology and regenerative medicine have enabled the use of cell-based therapy in periodontal diseases (reviewed in [5, 12]). To date, a large number of studies have indicated that ex vivo-manipulated stem cells derived from either bone marrow or the PDL can be used in conjunction with different physical matrices (autografts, xenografts, allografts, and alloplastic materials) to regenerate periodontal tissues in vivo (reviewed in [13–15]).

Although controversy remains regarding which tissues provide the most appropriate donor source for cell isolation, there is evidence that the cells of PDL tissues have the capacity to form a complete periodontal attachment apparatus (reviewed in [13–15]). The regenerative capacity of the PDL is attributed to a few progenitor cells within the PDL that maintain their proliferation and differentiation potential; thus, regeneration of the periodontium depends on the participation of these mesenchymal stem/stromal cells (MSCs) (reviewed in [5, 12, 13]). PDL-derived progenitors are committed to several developmental lineages, i.e., osteoblastic, fibroblastic and cementoblastic [16], which suggests that these cells are capable of regenerating multiple periodontal tissues. Indeed, positive preclinical results have been achieved in a wide range of in vitro and in vivo models [17–28]. The next phase of study requires the clinical application of these advanced therapies.

Worldwide, periodontitis remains highly prevalent and leads to a loss of the affected teeth. This disease threatens the quality of life of the middle-aged population as far as oral functioning is concerned. Unfortunately, no current clinical periodontal treatments can heal the defects in the affected region or regenerate lost periodontal tissue to a normal structure and functionality. It is clear that there is a clinical need for such treatments and a vast patient demand. Importantly, several groups have commenced small-scale pilot/feasibility studies in humans [21, 29–31]; thus, there is now sufficient information to support endeavors to move cell-based periodontal therapy into the clinical arena. We established a clinical protocol to further test the safety, feasibility, and potential efficacy of stem cells for the treatment of periodontal deep intraosseous defects.

## Methods

### Study design

**Fig. 1:**
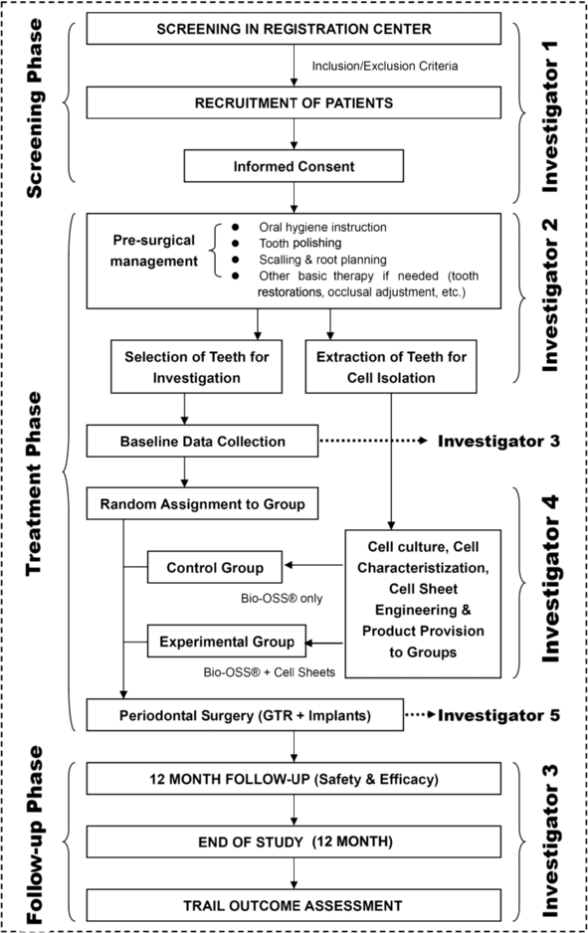
The flow chart of the trial describes the selection, randomization, treatment and follow-up process. A physician (Investigator 3) performed the follow-up examination of the patients and remained blinded to the treatment conditions until the clinical trial was completed. *GTR* Guided tissue regeneration

This study had a randomized design involving one dental facility (Translational Research Center, School of Stomatology, Fourth Military Medical University) and was conducted in compliance with Good Clinical Practice (GCP) guidelines according to the schedule shown in Fig. 1. This clinical trial, including the recruitment of subjects, was performed from 1 June 2011 to 30 December 2013, and the study was completed at the end of 2014 with a 1-year follow-up of the patients. The study protocol for this trial is provided in Additional file 1.

### Ethics

This study was approved by the ethical committees of the School of Stomatology, Fourth Military Medical University (2011-02) and is registered with the ClinicalTrials.gov database (reference no. NCT01357785). This study was conducted according to the Declaration of Helsinki, and all recruited patients consented to participate in this trial and contribute their trial data for noncommercial purposes. The protocol of this trial was externally reviewed and approved by an anonymous independent ethical review committee to ensure no serious ethical concerns.

### Patients, enrollment and randomization

Patients with periodontitis visiting our dental institution were requested to participate in the study. In compliance with GCP guidelines, prospective patients who provided written informed consent underwent clinical inspection and an oral cavity diagnosis. We selected subjects who satisfied the inclusion and exclusion criteria (recorded as the date of recruitment). The majority of these criteria were used in previous similar periodontal clinical trials [32, 33]. The inclusion and exclusion criteria and methods for randomization are provided in Additional file 2 (Appendices 1 and 2).

### Study products and interventions

**Fig. 2:**
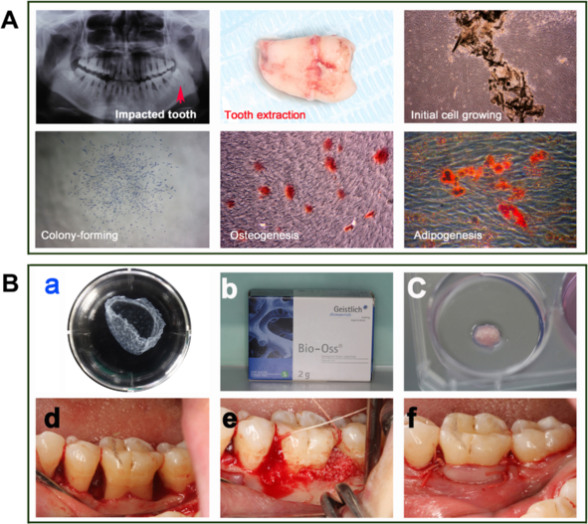
Cell isolation, characterization and surgery. **A** The impacted third molar of patients was extracted and subjected to cell isolation and cell characterization for cell colony-forming ability and osteogenic/adipogenic differentiation. **B** The production of cell sheet/scaffold transplants and in vivo transplantation, including: (*a*) cell sheet formation; (*b*) Bio-Oss^®^ particulates; (*c*) cell sheet/scaffold transplants; (*d*) exposure of bone defects; *(e*) placement of transplants; and (*f*) closure of the flap

The third molars of the patients in the Cell group were extracted and subjected to cell isolation and transplant production according to the Good Laboratory Practice and Good Manufacturing Practice (GMP) guidelines. The cells were assessed for cell colony-forming ability and osteogenic/adipogenic differentiation (Fig. 2A). Prior to the extraction surgery, at least two independent assessors concluded that a tooth (or teeth) extraction was required due to impacted or nonfunctional reasons. The methods for cell isolation and characterization are presented in Additional file 2 (Appendix 3). The PDL cell sheets obtained from the patient’s own tooth/teeth (see inclusion criteria) were produced using the Good Laboratory Practice and GMP guidelines using a standardized procedure in the Research and Development Center for Tissue Engineering (Fourth Military Medical University, 145th West Chang-le Road, Xi’an 710032, Shaanxi, People’s Republic of China). The detailed method is described in Additional file 2 (Appendix 3). Bio-Oss^®^ and Bio-Guide^®^ were purchased from Geistlich Pharma AG (Volhusen, Switzerland). Both transplants (Bio-oss^®^ only or Bio-oss^®^/cell sheets) were freshly prepared by laboratory researchers (Fig. 2Ba–c). Investigator 3, who performed the follow-up study, was kept blinded to the treatment conditions until the study was completed. For the surgical treatment, Bio-oss^®^ only (Control group) or Bio-oss^®^/cell sheets (Cell group) were administered only to the bony defect region (Fig. 2Bd–f). Each subject received a standard initial preparation, including oral hygiene instruction, full-mouth scaling, and root planning before surgical treatment, in order to minimize the bacterial insult and reduce variability between lesions at baseline. The operations were performed using GCP procedures. A 12-month postoperative follow-up was performed for each patient.

### Safety assessment

A cell safety assessment was performed to detect chromosomal karyotype changes between freshly isolated periodontal ligament stem cells (PDLSCs) and those obtained from the cell sheets (the latter underwent approximately 30-day ex-vivo cultures). The detailed methods are described in Additional file 2 (Appendix 3). Complications and adverse events during postoperative healing were recorded, and we examined the extent of adverse event occurrence. In addition, blood was obtained from patients preoperatively and at 2 weeks, 3 months and 12 months postoperatively. Blood examinations included: (i) a decrease in the white blood cell count; (ii) an increase in the red blood cell count; (iii) a decrease/increase in the percentage of neutrophils; (iv) a decrease/increase in the percentage of lymphocytes; (v) an increase in blood bilirubin; (vi) a decrease in blood lactate dehydrogenase; (vii) an increase in C-reactive protein; and (viii) an increase in creatinine phosphokinase. Moreover, the levels of IgA, IgG, IgM, C3 and C4 were measured in the serum using enzyme-linked immunosorbent assay (ELISA) at the Department of Clinical Laboratory, Fourth Military Medical University School of Stomatology. At the time of blood collection, the urine of each patient was collected and assessed for: (i) a positive test for glucose/albumin; (ii) an increase in β-N-acetyl-*D*-glucosaminidase; and (iii) an increase in β2 microglobulin.

### Efficacy assessment

The main outcome measure in the study protocol was the rate of increase in alveolar bone height at 3, 6 and 12 months postoperation (primary outcome). The bone-defect depth (the distance in millimeters from the deepest part of the defect to the cementoenamel junction of the tooth) was measured as described in Additional file 2 (Appendix 4) [34]. The clinical attachment level (CAL), probing depth (PD) and gingival recession (GR) measured in millimeters are generally used to assess pathology in periodontal disease. However, these parameters do not directly assess the efficacy of cells in periodontal tissue regeneration and were selected as secondary outcome measures to ascertain if the cells caused abnormal periodontal healing following periodontal surgery. The methods for the determination of these parameters at baseline and 3 months postoperation are described in Additional file 2 (Appendix 4) [32, 33].

### Statistics

This study was performed using a per-protocol analysis. In this analysis, all of the randomized teeth received at least one therapy, but the teeth that did not receive treatment were excluded (modified per-protocol analysis). The last-observation-carried-forward method was used for the per-protocol analysis. The missing data points were input into the postbaseline follow-up visits from the last observation available for each patient. For analysis, we employed SAS version 8.2 software (SAS Institute Inc., Carey, North Carolina, USA). The per-protocol set analysis was performed for the primary outcome. The baseline between-group comparisons in age and clinical examination indices were performed using independent group *t* tests. The between-group comparison of sex was performed using the Fisher’s exact probability test. The changes in clinical examination indices were tested using a repeated-measures analysis of variance. The level of statistical significance was set at *p* < 0.05 prior to analysis.

## Results

### Enrollment and teeth

**Fig. 3:**
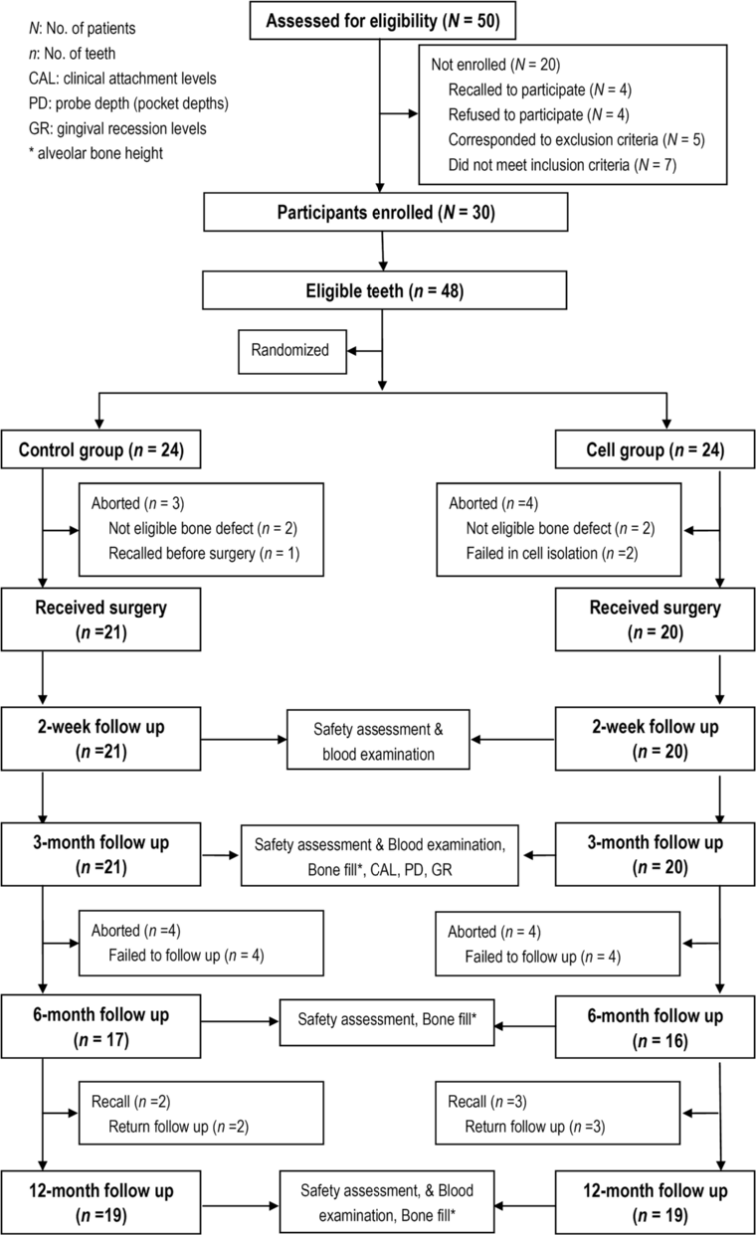
The flow diagram of the trial

The flow diagram for the study is shown in Fig. 3. A total of 48 screened teeth were randomly assigned to either the Control group or the Cell group. However, only 41 teeth received surgery (21 teeth in the Control group and 20 teeth in the Cell group). The baseline measurements of the teeth are shown in Table 1. A Fisher’s exact probability test found no significant between-group differences in the donors who provided teeth for randomization and testing.

**Table 1:** Teeth and baseline

	Control group	Cell group	*p* value
Sex (*n*)	21	20	0.134^a^
Male (*n*)	6	2	
Female (*n*)	15	18	
Age (years, mean ± SE)	30.04 ± 7.90	26.05 ± 4.44	0.053^b^
CAL (mm, mean ± SE)	5.28 ± 1.60	5.15 ± 1.52	0.795^b^
BDD (mm, mean ± SE)	7.19 ± 1.87	7.20 ± 2.65	0.990^b^
PD (mm, mean ± SE)			
Facial	5.68 ± 1.59	6.43 ± 1.92	0.185^b^
Lingua (palatal)	5. 86 ± 1.43	6.25 ± 1.36	0.373^b^
GR (mm, median (interquartile range))			
Facial	0.33 (1.0)		0.692^c^
Lingua (palatal)	0.33 (0.83)		0.320^c^

^a^Fisher’s exact probability test; ^b^independent group *t* test; ^c^Mann-Whitney test. *BDD* bone-defect depth, *CAL* clinical attachment levels, *GR* gingival recession, *PD* probe depth, *SE* standard error

### Cell culture and surgery

In this trial, patients who had at least one tooth (e.g., wisdom tooth) that needed to be extracted due to impaction or nonfunctional reasons and agreed to the tooth extraction were enrolled. Prior to extraction surgery, at least two independent assessors concluded that a tooth or teeth required extraction. The extracted teeth were used for cell isolation. Only two teeth failed during the cell isolation step, and the corresponding two patients were excluded from further study. All of the cells exhibited colony-forming ability. In addition, these cells were positive for the MSC markers STRO-1, CD146, CD105, CD29, and CD90 and negative for the hematopoietic markers CD31 and CD45. The cells were successfully differentiated in osteogenic and adipogenic microenvironments and subsequently used for cell sheet production and periodontal surgery (refer to Additional file 2 (Appendix 3) for more information).

### Safety evaluation

Postoperative healing occurred without significant problems, and none of the patients reported any complications/adverse events other than medium-sized swelling and pain. None of the pain experienced by patients required therapy. All of the patients underwent blood and urine tests preoperatively and at 2 weeks, 3 months and 12 months postoperatively. Changes in the white/red blood cell count, percentage of neutrophils/lymphocytes, and blood bilirubin/lactate dehydrogenase/C-reactive protein/creatinine phosphokinase levels were within the clinically accepted range (no measurement exceeded its clinical reference value). Importantly, no significant changes in IgA, IgG, IgM, C3 or C4 concentrations were found in the serum of any of the patients. A urine test showed that one patient (with one tooth that received GTR and Bio-oss^®^ therapy without stem cells) was positive for glucose (this patient was ultimately not diagnosed with diabetes mellitus) and two patients were positive for albumin (each patient had two teeth involved in this trial, and one tooth per patient received cell therapy). No significant changes in urinary β-N-acetyl-*D*-glucosaminidase or β2 microglobulin were found for any of the patients.

### Evaluation of efficacy

**Fig. 4:**
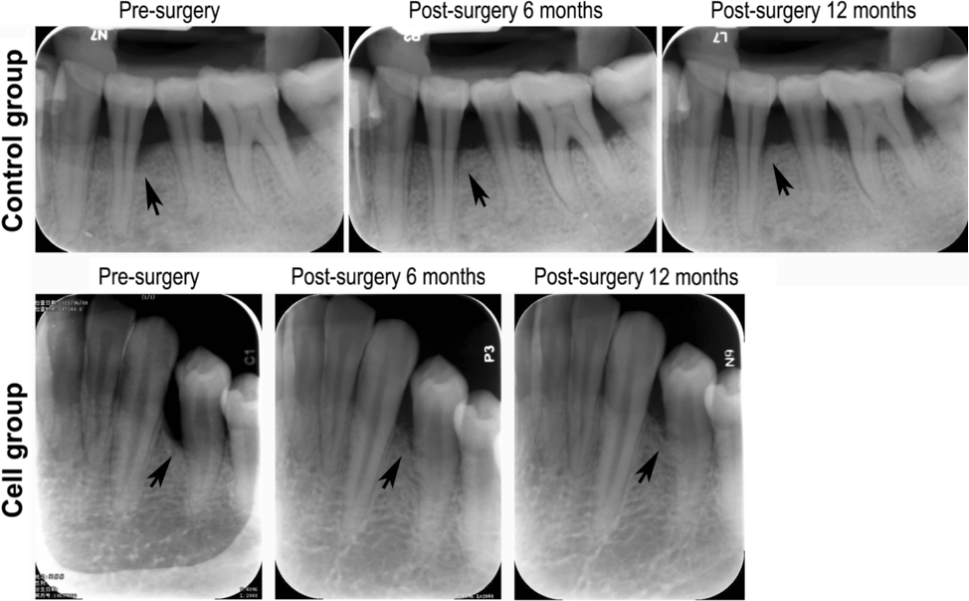
Radiographic evidence for bone height increases in the Control group and the Cell group (*black arrowheads* points to sites of bone defect in each radiograph)

Patient demographic data and the baseline measurements of the affected teeth are shown in Table 1. All of the treated teeth in both groups adequately recovered following surgery. There was no loss of treated teeth during this trial. X-ray examinations showed significant bone fill in both groups (Fig. 4). The magnitude of increase in alveolar bone height at 3, 6 and 12 months (bone fill over time) was determined as the decrease in the bone-defect depth. Each group showed a significant increase in the alveolar bone height over time (*p* < 0.001). However, no statistically significant differences were found between the Cell group and the Control group (*p* > 0.05) (Table 2). Regarding the clinical periodontal parameters, no statistically significant differences were found for the increased CAL, PD or GR between the Cell and Control groups at 3 months postsurgery (*p* > 0.05) (Table 3).

**Table 2:** Bone-defect depth with time (the distance from the deepest part of the defect to the cementoenamel junction of the tooth in mm, mean ± standard error)

	No. of teeth	Baseline	3 months	6 months	12 months	*F* value	*p* value
Control group	21	7.19 ± 1.87	4.81 ± 1.93	5.11 ± 1.53	4.80 ± 1.41	0.11	0.742^a^
Cell group	20	7.20 ± 2.65	4.89 ± 1.73	4.61 ± 1.87	4.49 ± 2.03		

^a^Repeated-measures analysis of variance

**Table 3:** Changes in clinical examination indices over time (mm, mean ± standard error)

	No. of teeth		Baseline	3 months	*F* value	*p* value
CAL						
Control group	21		5.28 ± 1.60	5.07 ± 1.48	0.817	0.371^a^
Cell group	20		5.15 ± 1.52	4.42 ± 1.19		
PD						
Control group	21	Buccal	5.68 ± 1.59	3.88 ± 0.77	0.962	0.333^a^
Cell group	20	Buccal	6.43 ± 1.92	3.80 ± 1.03		
Control group	21	Lingual or palatal	5. 86 ± 1.43	3.79 ± 0.55	2.191	0.147^a^
Cell group	20	Lingual or palatal	6.25 ± 1.36	4.20 ± 0.86		
GR						
Control group	21	Buccal	0.62 ± 0.89	1.54 ± 0.96	0.133	0.728^a^
Cell group	20	Buccal	0.70 ± 1.09	1.28 ± 0.82		
Control group	21	Lingual or palatal	0.52 ± 0.85	1.38 ± 1.37	0.012	0.915^a^
Cell group	20	Lingual or palatal	0.73 ± 0.87	1.23 ± 0.92		

^a^Repeated-measures analysis of variance. *CAL* clinical attachment levels, *PD* probe depth, *GR* gingival recession

## Discussion

Although there are a number of clinical techniques available for the management of periodontal intrabony defects, clinicians continue to seek more predictable regenerative therapies that are less technique-sensitive, lead to rapid tissue regeneration, and applicable to the broad array of periodontal conditions that are encountered daily in the clinic. Recent evidence from animal models [17–28] and several small-scale pilot/feasibility studies [21, 29–31] indicates that ex vivo*-*cultured PDL cells may serve as a powerful tool for periodontal therapy. A number of animal studies have provided an overwhelming body of evidence that MSCs can be safely and effectively used for periodontal regeneration (reviewed in [12]). As a consequence of these successful animal studies, the clinical application of stem cells for the regeneration of periodontal tissue has begun [30, 31].

Substantial evidence suggests that it is time is to move cell-based periodontal therapy from animal studies to human clinical trials. However, there are critical steps in moving this field towards human clinical utility. In addition to clinical efficacy, the safety of cell-based therapies has not been fully evaluated, and the risks of stem cell therapies have been underscored by several clinicians and researchers. Moreover, issues, such as cell delivery, cell immunogenicity, use of autologous cells or allogeneic cells, control of cell fates in vitro and in vivo, and cost-effectiveness, are all important considerations that should be addressed before this therapy can move forward [35–39]. The next critical phase requires the identification of tissues that provide the most appropriate donor source(s) and the systematic validation of these specific MSCs as reliable for periodontal cytotherapeutic use. Furthermore, the establishment of large-scale preparation facilities incorporating the stringent protocols of GMP will be an absolute necessity. Regulatory agencies need to define new criteria to evaluate the risk associated with specific stem cells and their differentiated progeny (reviewed in [35, 36]). The purpose of this trial was to provide evidence for the use of ex vivo-cultured cells to treat periodontitis and determine the best approach to treat this disease.

For incurable and life-threatening diseases, such as diabetes, Parkinson’s, muscular dystrophy, Alzheimer’s, neural and cardiac diseases and refractory systemic lupus erythematosus, cell-based therapy is more likely to be warranted and accepted by the government and patients [37–39]. However, periodontal tissue regeneration using cell therapy is not yet economically viable or competitive with current root canal therapies and dental implants. Due to the non-life threatening nature of periodontitis, periodontal tissues have not been considered to be a major target for stem cell-based regenerative medical research. Nevertheless, affected teeth are ideal for the evaluation of new therapies because the patients are not usually ill. Thus, if anything goes wrong with the treatment, the situation is far less likely to be life threatening. Furthermore, the accessibility of teeth facilitates treatments that do not require major surgery [40].

In a previous study by Feng et al., periodontitis patients were treated with the local administration of autologous PDL or gingival stem cells. Neither adverse reactions nor an increase in any autoantibodies was observed; however, only three cases were observed long-term [30]. To move cell-based periodontal therapy from preclinical study and case report into clinical trials, we conducted this study to evaluate the safety and primary efficacy of this treatment. The results of this study have implications for oral health care resourcing and may facilitate improvements in the treatment of people with periodontitis. No clinical safety problems attributable to the investigational PDLSCs were identified in our study. Each group showed a significant increase in alveolar bone height over time (*p* < 0.001; Table 2). Although no statistically significant differences were found for the outcomes in the bone and clinical parameters between the Cell and Control groups (*p* > 0.05; Tables 2 and 3), the results of this study were the first to show that cell-based interventions are safe for human dental use in trials. Geistlich Bio-Oss^®^ is a natural bone substitute with osteoconductive properties; however, there is no substantial evidence that this product will lead to effective or predictable bone regeneration in periodontal intrabony defects. Because Bio-Oss^®^ particles may become an integral part of the newly formed bone framework and preserve volume over time [41], the use of this material as a scaffold for PDLSCs requires further investigation. Previous studies showed that allogeneic bone matrix may regenerate new bone, new cementum, and a new PDL around teeth that were previously contaminated by bacterial plaque [42, 43]. Human-derived biomaterials would be the first choice as a cell carrier in similar future trials [44]. Nevertheless, the results that were obtained in this study may have important implications for the design of more appropriate cell-delivery materials to test cell therapies in patients with periodontitis.

The study presented here used central randomization, which is a strict and complete randomization method that ensures adequate concealment. The surgeon and the investigator who collected the baseline and follow-up data worked independently in this trial. Throughout the entire trial, the patients were not aware of which group they were assigned to. The patients were only informed that they would receive a periodontal surgical treatment that potentially included cell products. This trial was a single-center, randomized controlled study of 30 patients with 12 months of follow-up. In a phase I clinical trial, safety assessments should be the primary outcome. The methodology in this study was designed to assess both safety and efficacy because the use of PDL-derived cells for periodontal therapy was previously reported several years ago [30]. Indeed, several groups worldwide have completed small-scale pilot/feasibility studies that indicated no adverse reactions after local cell administration [29, 31]. Although these studies did not have a randomized designed, the reported safety of the treatment offers substantial evidence to justify the use of cell-based periodontal therapy in the clinic. To date, this study was one of the largest randomized controlled trials addressing the safety and effectiveness of cell therapy in combination with GTR and bone replacement for periodontitis. However, this study does not deliver substantial evidence to confirm the safety of cell-based periodontal therapy. Similar to the clinical use of stem cells in the oral cavity (excluding the periodontium) for bone regeneration [45, 46], cell-based techniques in periodontal regenerative medicine should be further investigated in more challenging clinical scenarios with well-designed and standardized randomized controlled trials. In addition, these therapies should be tested in combination with bioactive molecules and new materials in an attempt to improve the final outcome. We plan to design a phase II clinical trial for cell-based periodontal therapy aimed at selecting more suitable scaffolding materials, determining appropriate cell doses, and providing evidence for multicenter, randomized controlled trials.

## Conclusions

Stem cell therapy is a promising new therapeutic avenue that may enable the regeneration of lost periodontal tissue, and regenerative dentistry is at the forefront of the transition from basic science research to the clinical reconstructive arena. Although there are many issues that need to be resolved before stem cell therapies become commonplace, clinicians should continue to monitor the progression of these technologies. The data obtained in this study showed that autologous PDLSC-based treatment for periodontal intrabony defects was safe; however, more rigorous clinical trials are recommended to evaluate the efficacy of this therapy. Future clinical endeavors in cell-based periodontal therapy should identify more suitable scaffolding materials and define safe and effective cell dosing procedures based on well-designed, multicenter, randomized controlled trials.

### Consent to publish

The authors confirm that they have obtained consent from the participants to publish the trial data.

## Additional files

Additional file 1:
**The study protocol (2011-06) for the trial.** (DOC 6959 kb) Additional file 2:
**Appendices (inclusion and exclusion criteria of the trial; detailed methods for randomization; detailed methods for cell isolation, characterization and cell transplant preparation; and detailed methods for determining bone fill).** (DOCX 2833 kb)

## Abbreviations

CAL: Clinical attachment level
GCP: Good Clinical Practice
GMP: Good Manufacturing Practice
GR: Gingival recession
GTR: Guided tissue regeneration
MSC: Mesenchymal stem/stromal cell
PD: Probing depth
PDL: Periodontal ligament
PDLSC: Periodontal ligament stem cell
