# Correction to: Treatment of periodontal intrabony defects using autologous periodontal ligament stem cells: a randomized clinical trial

**DOI:** 10.1186/s13287-018-1000-4

**Published:** 2018-10-07

**Authors:** Fa-Ming Chen, Li-Na Gao, Bei-Min Tian, Xi-Yu Zhang, Yong-Jie Zhang, Guang-Ying Dong, Hong Lu, Qing Chu, Jie Xu, Yang Yu, Rui-Xin Wu, Yuan Yin, Songtao Shi, Yan Jin

**Affiliations:** 1 State Key Laboratory of Military Stomatology, Department of Periodontology, School of Stomatology, Fourth Military Medical University, Xi’an, Shannxi People’s Republic of China; 2 State Key Laboratory of Military Stomatology, Research and Development Center for Tissue Engineering, School of Stomatology, Fourth Military Medical University, Xi’an, Shannxi People’s Republic of China; 3 Department of Anatomy and Cell Biology, School of Dental Medicine, University of Pennsylvania, 240 South 40th Street, Philadelphia, PA 19104 USA

## Correction

The original article [1] contains a major error carried across the captions of Tables 1, 2, and 3. In each table caption, the data were expressed as “mean ± standard deviation (SD)”; unfortunately, the authors had mistakenly expressed the data as “mean ± standard error (SE)” instead. As such, all mentions of “mean ± standard error” in those table captions should of course state “mean ± standard deviation”. The authors are deeply sorry for these errors.
