# Supplementary material for: Treatment of periodontal intrabony defects using autologous periodontal ligament stem cells: a randomized clinical trial
Source: Stem Cell Res Ther. 2016 Feb 19;7:33. doi: 10.1186/s13287-016-0288-1 (PMC4761216; doi:10.1186/s13287-016-0288-1)
Supplement: Additional file 1: — The study protocol (2011-06) for the trial. (DOC 6959 kb) [file 13287_2016_288_MOESM1_ESM.doc]

***Additional file 1***

**Study protocol**

**(Trial Registration: NCT01357785)**

**Treatment of periodontal intrabony defects using autologous periodontal ligament stem cells: A randomized clinical trial**

**Fa-Ming Chen**

**Yong-Jie Zhang**

**Yan Jin**

**Songtao Shi**

**Translational Research Team, School of Stomatology**

**Fourth Military Medical University**

**2011.06**

**Abstract**

**Background**: Periodontitis, which progressively destroys tooth-supporting structures, is one of the most widespread infectious diseases and the leading cause of tooth loss in adults. Evidence from animal studies indicates that stem cells derived from either bone marrow or the periodontal ligament (PDL) can be used in therapies for the regeneration of lost or damaged periodontal tissues. The aim of this clinical trial is to identify the safety of the clinical use of stem cells in periodontal therapy and to verify whether stem cells in combination with Bio-oss® are more effective than Bio-oss® alone in guided tissue regeneration (GTR) techniques for treating periodontal disease in human patients.

**Methods/Design**: In this protocol, we designed a single-center randomized trial to treat periodontal disease using GTR techniques and bovine-derived bone mineral (BDBM, Bio-oss®) with or without the incorporation of autologous periodontal ligament stem cells (PDLSCs). In total, approximately 30 periodontitis patients aged 18 to 65 years with a total of approximately 40 test teeth with periodontal intrabony defects and who satisfy our selection and exclusion criteria will be enrolled. These patients will be randomly assigned to either the Cell testing group (treatment with GTR and PDLSC sheets in combination with Bio-oss®) or the Control group (treatment with GTR and Bio-oss® without containing cells). During a 12-month follow-up, we will examine the extent of any adverse events related to the use of PDL cells. Moreover, the primary outcome is the magnitude of change in the alveolar bone height, and the secondary outcome measures are improvements in clinical attachment level (CAL), probing depth (PD), and gingival recession (GR).

**Discussion**: The results of this trial will assess the efficacy, safety and feasibility of using autologous PDLSCs to treat periodontal intrabony defects, and these data will provide primary clinical evidence for the future use of autologous stem cells in regenerative periodontal therapy.

**Background**

Periodontitis is an inflammatory disease that causes pathological alterations in tooth-supporting tissues and can lead to tooth loss if left untreated. National surveys have shown that the majority of adults suffer from moderate periodontitis, and up to 15% of the population is affected by severe generalized periodontitis at some stage of their lives [1,2]. The significant burden of periodontal disease and its impact on the general health and quality of life of the patient indicates a clinical need for the effective management of this condition [3]. Notably, the ultimate goal of periodontal therapy is the predictable regeneration of the functional attachment apparatus destroyed by periodontitis, which involves at least three unique tissues, *e.g.,* cementum, the periodontal ligament (PDL), and alveolar bone. To date, several regenerative procedures have attempted to achieve this ambitious goal, including guided tissue regeneration (GTR), bone graft placement, and the use of bioactive agents, such as growth factors (reviewed in [4-8]). However, current therapeutic techniques that are used either alone or in combination are limited in their ability to attain complete and predicable regeneration, especially in advanced periodontal defects. In these cases, remaining deep intraosseous defects following periodontal therapy have been shown to be high-risk sites for the further progression of periodontitis (reviewed in [9-12]).

According to the histological evidence, the GTR technique in combination with grafting materials, such as Bio-oss® (Geistlich Pharma AG, Volhusen, Switzerland) and autologous bone, is partially effective at treating periodontal defects; however, the currently available GTR-based therapies are rudimentary and show poor clinical predictability [4,5,8]. Recent advances in stem cell biology and regenerative medicine have enabled the use of cell-based therapies in periodontal diseases [8,16]. To date, a large number of animal studies have indicated that *ex vivo*-manipulated stem cells derived from either bone marrow or the periodontal ligament (PDL) can be used in conjunction with different physical matrices to regenerate periodontal tissues *in vivo* (reviewed in [7,8,13-19]).

Although controversy remains regarding which tissues provide the most appropriate donor source for cell isolation, there is evidence that the cells of PDL tissues have the capacity to form a new and complete periodontal attachment apparatus (reviewed in [14-17]). The regenerative capacity of the PDL is attributed to a few progenitor cells within the PDL that maintain their proliferation and differentiation potential; thus, regeneration of the periodontium depends on the participation of these mesenchymal stem/stromal cells (MSCs) (reviewed in [14,15]). PDL-derived progenitors are committed to several developmental lineages, i.e., osteoblastic, fibroblastic and cementoblastic, which suggests that PDL-derived cells are capable of regenerating a functional periodontal attachment apparatus. Indeed, positive pre-clinical results have been achieved in both *in vitro* and *in vivo* models [20-29]. The next phase of study requires the clinical application of these advanced therapies.

Worldwide, periodontitis remains highly prevalent, can cause the loss of affected teeth. This disease threatens the quality of life for the middle-aged population as far as oral functioning is concerned. Unfortunately, the best currently available periodontal treatments heal with a scar in the affected region and have less capacity to regenerate lost periodontal tissue to a normal structure and functionality. Considering that the mouth and teeth have various aesthetic and functional roles to play, establishing a new and innovative treatment that enables the predictable regeneration of periodontal tissue is very important. It is clear that there is a clinical need and a large patient population for these treatments.

A previous study by Drs. Feng and Shi indicated that systemically healthy male patients with periodontitis who were treated by the local administration of autologous PDL and gingival stem cells showed no adverse reactions. However, only part of this study was published [30]. Importantly, several other groups around the world have completed small-scale pilot/feasibility studies [31-33] that provide sufficient information to shift cell-based periodontal therapy into the clinical arena. Therefore, we established this clinical protocol to test the efficiency and safety of stem cells as a treatment for periodontal deep intraosseous defects.

**Methods/Design**

**Overview**

***Purpose***

The purpose of this clinical trial is to confirm the safety of autologous stem cells in clinical periodontal regenerative surgery and clarify the efficiency of autologous periodontal ligament (PDL) stem cells to form cell sheets that can be used to regenerate periodontal tissue in periodontitis patients with deep intraosseous defects (>5 mm). This study is a single-center, randomized, controlled study (Figure 1) that was approved by the ethical committees of School of Stomatology, Fourth Military Medical University (2011-02) and registered in the ClinicalTrials.gov database (reference no. NCT01357785). The study will be conducted according to the Declaration of Helsinki, and we will obtain consent from all enrolled subjects with regards to participation in this trial and contribution of trial data for non-commercial purposes. The protocol of this trial was externally reviewed and approved by an anonymous independent ethical review committee to ensure no serious ethical concerns. This study is a randomized clinical trial that includes a test and control group for comparison and involves only one dental facility (Translational Research Team, School of Stomatology, Fourth Military Medical University). The clinical trial will be conducted according to the schedule shown in Figure 1.

***Main outcome measures***

In this study, we will examine the extent of adverse events related to the use of PDL cells. In addition to the safety assessment, the main outcome measure in the study protocol is the magnitude of change in the alveolar bone height (primary outcome for efficacy).

**Study Design**

Each patient will be examined by at least five independent principal investigators. Investigator 1 will perform a basic oral examination using a clinical inspecting apparatus and X-ray, check all of the inclusion and exclusion criteria, and ensure that the informed consents are signed by the patients at the Registration Center. Next, Investigator 2 will perform the pre-surgical treatments, re-check the inclusion and exclusion criteria, and extract the tooth/teeth that will be subjected to cell isolation by Investigator 4. Investigator 2 will select no more than 2 teeth (from different regions) to be investigated in each patient. Following the collection of baseline data from the selected tooth/teeth by Investigator 3, Investigator 4 will culture the cells, make the cell products, and create an allocation table consisting of 2 cases per block that will be allocated to the Control group (Bio-oss® only) and Cell group (Bio-oss® and cell sheets). According to this allocation table, a label indicating the corresponding group will be attached to each tooth; thus, each tooth will be randomly assigned to one of the two treatment groups. The maximum number of teeth from one patient will be 2. Hence, if one tooth is allocated to the Control group, the other will be automatically assigned to the Cell group. However, if a patient only has one tooth chosen for investigation, it has a fair chance of being allocated to either group. Once a tooth is assigned to a group, it will be kept in a secure locked drawer inaccessible to all other study personnel. All of the patients will receive oral hygiene instructions, a tooth cleaning and basic dental therapies if needed, such as cavity filling and occlusal adjustment. The periodontal surgery will be performed 4-5 weeks after these pre-surgical treatments. When the oral condition and cell products are both ready for surgery, Investigator 4 will be asked to give the assigned implants (Bio-oss® only or a combination of Bio-oss® and cell sheets) to the physician (Investigator 5), who will then perform the surgery. This procedure ensures that the randomization is not influenced by the physicians taking part in this study. Investigator 4 will record the patients’ details and number the envelope assigned to each tooth. Investigator 3 will perform the follow-up study (data collection, safety assessment, etc.) of the patient, and the blind will not be broken until this clinical trial is completed.

***Study Products***

Bio-Oss® and Bio-Guide® (Geistlich Pharma AG, Volhusen, Switzerland) and PDL cell sheets (cells obtained from a patient’s tooth/teeth; see inclusion criteria) will be produced at the Research and Development Center for Tissue Engineering (Fourth Military Medical University, 145th West Chang-le Road, Xi’an 710032, Shaanxi, People’s Republic of China) using a standardized procedure (see section 5.1. cell products). Both transplants (Bio-oss® only or Bio-oss®/cell sheets) will be prepared (ready for immediate use) by the laboratory researchers, and Investigator 3, who will perform the follow-up study, will remain blinded to the treatment conditions.

***Duration of the Study***

This clinical trial, including the recruitment of subjects, will be performed from June 1, 2011 to December 30, 2013, and the study is anticipated to finish at the end of 2014.


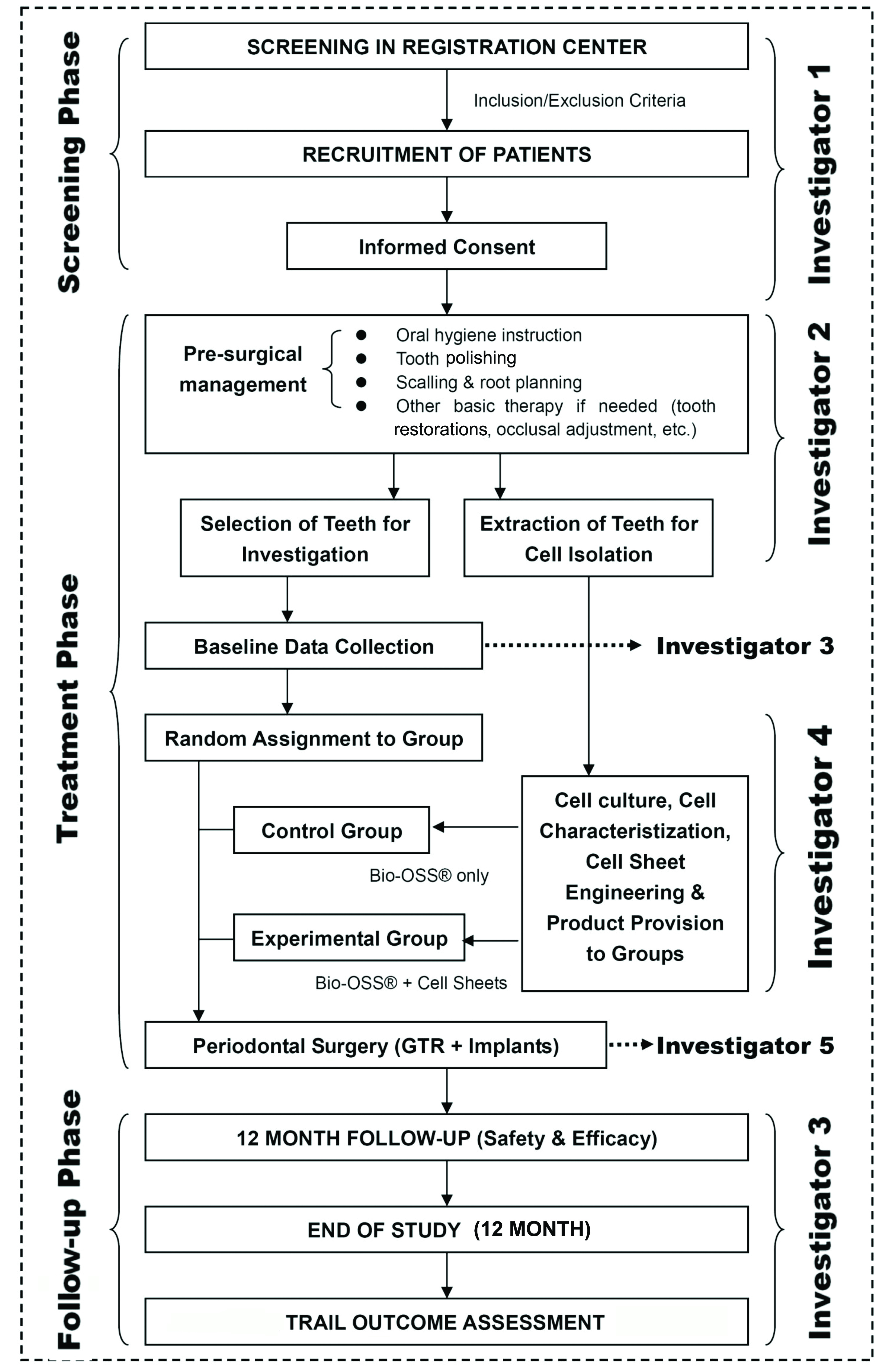


**Figure 1**. Flow chart of the trial describing the selection, randomization, treatment and the follow-up process that will be completed by the clinician (Investigator 3).

**Patients and enrolment**

Patients with periodontitis visiting our dental institution will be requested to participate. This study will be conducted in compliance with Good Clinical Practice guidelines. Prospective patients who provide written informed consent will undergo clinical inspection and an oral cavity diagnosis. We will then select approximately 30 patients (approximately 40 testing teeth) who satisfy the selection and exclusion criteria described below (most of these criteria were chosen from previous similar periodontal clinical trials [34,35]).

***Inclusion criteria***

- those ≥18 years old and <65 years old;
- those diagnosed with a 2- or 3-walled vertical intrabony defect ≥3 mm deep from the top of the remaining alveolar bone from radiography and clinical periodontal parameters;
- those who have accomplished the initial preparation and shown good compliance;
- those with a mobility of Degree 2 or less for the tooth under investigation and with a width of the attached gingiva for which the existing GTR and bone graft implantation treatments are considered appropriate;
- those for whom supportive periodontal treatment is applicable (the tooth has the potential to be maintained for at least 3 years) and in accordance with the usual post-operative procedures following flap operation and GTR treatment;
- those whose oral hygiene is well established and who are able to perform appropriate tooth brushing and cleaning following instructions from the investigators and/or sub-investigators after investigational drug administration;
- those who have at least one tooth (e.g., wisdom tooth) that needs to be extracted due to impacted or non-functional reasons and who agree to the tooth extraction;
- those who understand the purposes of the trial and are capable of making an independent decision to comply with trial requirements.

***Exclusion criteria***

- those who took a calcium antagonist during the 4 weeks prior to surgery;
- those in need of the administration of adrenal cortical steroids (equivalent to >20 mg/day of Predonin) within 4 weeks after the surgery;
- those scheduled to undergo a surgical operation in the vicinity of the tooth to be investigated within 36 weeks after surgery;
- those with coexisting mental or consciousness disorders;
- those with coexisting malignant tumors or history of the same;
- those with coexisting diabetes (HbA_1C_ >6.5%);
- those in extremely poor nutritional condition (serum albumin concentration <2 g/dL);
- those with ≥200 mL of blood drawn during the 4 weeks prior to surgery;
- those given another investigational drug during the 24 h preceding our investigational drug administration;
- those with a coexisting disorder of the kidney, liver, blood and/or circulatory system (Grade 2 or above);
- those who are either pregnant, possibly pregnant or breast-feeding or who hope to become pregnant during the trial period;
- those with a previous history of hypersensitivity to any biologically active drugs;
- those who smoked more than 10 cigarettes during the past 3 months;
- those who are involved with the research team of this trial;
- those with any other condition, as determined by the investigators or sub-investigators, that could make the subject unsuitable for the trial, impair the validity of the informed consent, or impair the subject's suitability for the trial.

**Cell products and interventions**

***Cell isolation***

Cells will be primarily cultured by an enzyme digestion procedure of the explantation from patient’s own PDL tissue from the third molar using an aseptic technique. The extracted teeth will be rinsed with α-minimum essential medium (α-MEM, Invitrogen, Carlsbad, CA) containing 100 units/mL penicillin and 100 mg/mL streptomycin (Invitrogen) for 3 min for a total of 5 times. Next, the PDL will be gently separated from the surface of the root and digested in a solution of 0.2% collagenase type I (sigma) for 15 min at 37°C. Single cell suspensions will be obtained by passing the sample through an 80-mesh strainer, deposition at 1000 r/min for 8 minutes, and then blown in α-MEM supplemented with 10% fetal bovine serum (FBS). After cell counting, the cultures will be inoculated in 25-cm^2^ culture flasks with 4 mL of α-MEM medium at a concentration of 5×10^5^ per flask and incubated in 5% CO_2_/95% air at 37°C. The culture medium will be changed once every two days until the cells grow to 80% confluence (7-10 days), at which time the cells will be passaged.

***Passage of cells***

After washing with phosphate buffered saline (PBS, Grand Island, NY, USA) to remove the remaining culture medium in the culture flask, the cells will be digested with 0.25% trypsin for 2 min at 37°C. α-MEM supplemented with 10% fetal bovine serum will be added to stop the digestion until the cells become circular and the majority are floating (observation made under reverse microscope). Cells will then be deposited at 800-1000 r/min for 6 minutes. Following the re-suspension of cells into PBS, a unicellular suspension will be used to inoculate the culture flask at a concentration of 5×10^5^/mL. The cell type, passage, cell density, date and operators will be noted. The amount of cells at passage 4 will be 1×10^7^ (passage time: approximately 20 days). The obtained cells will be identified by adipogenic and osteogenic differentiation.

To analyze the surface antigens, PDLSCs will be washed in PBS and then incubated with 2 μg/mL of the following mouse anti-human monoclonal antibodies: CD29, CD44, CD73, CD90, CD105, CD34, CD45, STRO-1 and CD146 for 30 min at 4 °C. To induce osteoblastic differentiation, 10^5^ PDLSCs will be seeded in 6-well plates and cultured. When the cells reach subconfluence, the culture medium will be changed to DMEM containing ascorbic acid (L-ascorbic acid 2-phosphate) (50 μg/mL) (Sigma–Aldrich), dexamethasone (10^−8^ M) (Sigma–Aldrich) and β-glycerophosphate (10 mM) (Sigma–Aldrich). The culture medium will be changed every 3 days for either 14 or 28 days. For Alizarin Red staining, the cells will be fixed with 70% ethanol for 15 min at room temperature and stained using a 1% Alizarin Red solution for 5 min at room temperature. After staining, the culture plate will be washed with tap water and air-dried.

To induce adipocyte differentiation, 10^5^ PDLSCs will be seeded in 6-well plates and cultured in growth medium. Adipocyte differentiation will be induced using adipocyte differentiation medium containing 1 μmol/L dexamethasone, 0.5 mmol/L IBMX, 10 μg/mL insulin, and 100 mmol/L Indomethacin until day 14. Next, the cells will be fixed using 70% ethanol for 15 min and washed twice with PBS. Oil Red O solution (Sigma) will then be added for 15 min, followed by rinsing of the plate with PBS.

***Cell sheets***

To create the cell sheet, the PDLSCs will be digested by trypsin to obtain single cell suspensions and then inoculated on 6-well plates at 1×10^5^ per well with L-ascorbic acid (vitamin C; VC, 30 μg/mL, Sigma) until confluent (approximately day 10). Next, we will observe changes in cell morphology and sheet-forming capacity. After a 10-day culture, white membranous substances will appear on the bottom of the wells. Before clinical application, the culture medium will be discarded, and the cell sheets will be rinsed twice with phosphate-buffered saline (PBS; Gibco). Next, the 0.2-µm CBB particulates will be distributed onto the surface of the cell sheets at a concentration of 0.25 g per well. The PDLSC cell sheets will then be rolled up to pack the Bio-oss® particulates for clinical treatment.

***Interventions***

The surgical treatment will be performed in the region chosen for investigation. The Bio-oss® only (Control group) or Bio-oss®/cell sheets (Cell group) will be administered to the bone defect region. Each subject will receive a standard initial preparation prior to the surgical treatment, including oral hygiene instruction, full-mouth scaling, and root planning, to minimize the bacterial insult and reduce the variability between lesions at baseline.

**Safety assessment**

***Observation of subjective symptoms and objective findings***

Medical findings for both the oral cavity and whole body will be confirmed by interview and visual inspection.

***Clinical inspections***

The inspection items that will be measured and evaluated are shown below. In cases where we discover unusual changes in any of the clinical inspection values within 4 weeks after the administration of cells, a follow-up survey will be conducted.

- Hematological test (2 mL of blood)

Red blood cell count, white blood cell count, hemoglobin, hematocrit, platelet count, differential counts of leukocytes (neutrophils, eosinophils, basophils, lymphocytes, and monocytes)

- Biochemical blood test (2 mL of serum)

Total protein, albumin, blood urea nitrogen, creatinine, uric acid, total cholesterol, total bilirubin, aspartate aminotransferase, alanine aminotransferase, alkaline phosphatase, lactic dehydrogenase, C-reactive protein (CRP), creatine kinase (CK), Na, K, and Cl

- Urinalysis

Qualitative (10 mL of urine): protein, sugar, urobilinogen

Quantitative (3.5 mL of urine): urinary albumin (in creatinine equivalents), N-acetyl-beta-D-glucosamidase (NAG), beta2-microglobulin

- Blood glucose control index test (2 mL of blood): Conducted only before registration

Hemoglobin A_1C_

Pregnancy test (1 mL of urine): Conducted only before registration

Chorionic gonadotropin

***Measurement of antibody levels in serum***

The Department of Clinical Laboratory, Fourth Military Medical University School of Stomatology, will measure the levels of IgA, IgG, IgM, C3 and C4 in serum using ELISAs.

**Adverse events**

***Definition***

An adverse event is any unfavorable and unintended sign (including an abnormal laboratory finding), symptom, or disease that is temporally associated with the use of the investigational cells whether or not it is considered to be related to the investigational cells. We will examine the extent of any adverse events.

***Evaluation of adverse events***

The investigators will record any adverse event with the exact name, onset date, severity, provided treatment and date of disappearance.

Each adverse event will be graded for severity using the following scale and criteria as outlined by the Ministry of Health and Welfare of the Chinese Government.

- **Mild**: The subject can continue the subsequent schedule without any treatment.
- **Moderate**: The subject can continue the subsequent schedule with treatment.
- **Severe**: The subject cannot continue the subsequent schedule.

The investigators will determine the relationship of the adverse event and the investigational cells.

The investigators will determine whether or not the adverse event is classified as a serious adverse event. A serious adverse event is defined as any experience suggesting a significant hazard, contraindication, side effect, or precaution. A serious adverse event includes any adverse experience resulting in any of the following outcomes:

- Death
- A life-threatening adverse experience
- Impatient hospitalization or prolongation of existing hospitalization
- A disability/incapacity that may interfere with the subject's daily life
- An adverse experience leading to a disability/incapacity
- A serious adverse experience at the same level as the above conditions
- A congenital anomaly/birth

**Efficacy assessment** [34,35]

The main outcome measure in the study protocol is change in the alveolar bone height at 3, 6, and 12 months post-operation. We set the rate of increase in alveolar bone height as the most statistically important outcome (primary outcome). The clinical attachment level (CAL), probing depth (PD), and gingival recession (GR) are generally used to assess pathology in periodontal disease. These parameters do not directly assess the efficacy of cells in periodontal tissue regeneration and were selected in the present study as secondary outcome measures to ascertain whether cells would cause abnormal periodontal healing following periodontal surgery. These measurements will be recorded at baseline and 12-months post-surgery.

***Standardized radiography and dental CT for regions of investigation*** [34,35]

Our geometrically standardized radiography employs dental film (Kodak InSight Super Poly-Soft; Eastman Kodak Company, New York, USA) and photograph indicators (Cone Indicator-II; Hanshin Technical Laboratory, Hyogo, Japan) that are customized with resin stents. Two doctors specializing in dental radiology from the Department of Oral Diagnosis at Fourth Military Medical University School of Stomatology will measure the changes in the alveolar bone height (%) using the methods described below (Figure 2). Each examiner blinded to the treatment conditions will independently measure this value, and the median of 2 measurements taken from the same image will be selected for analysis. In addition, a dental CT that reports data automatically will be used to assess bone regeneration in selected cases. These data will be used for information purposes only or as a reference for the design of subsequent trials (not included in the analysis of this trial).


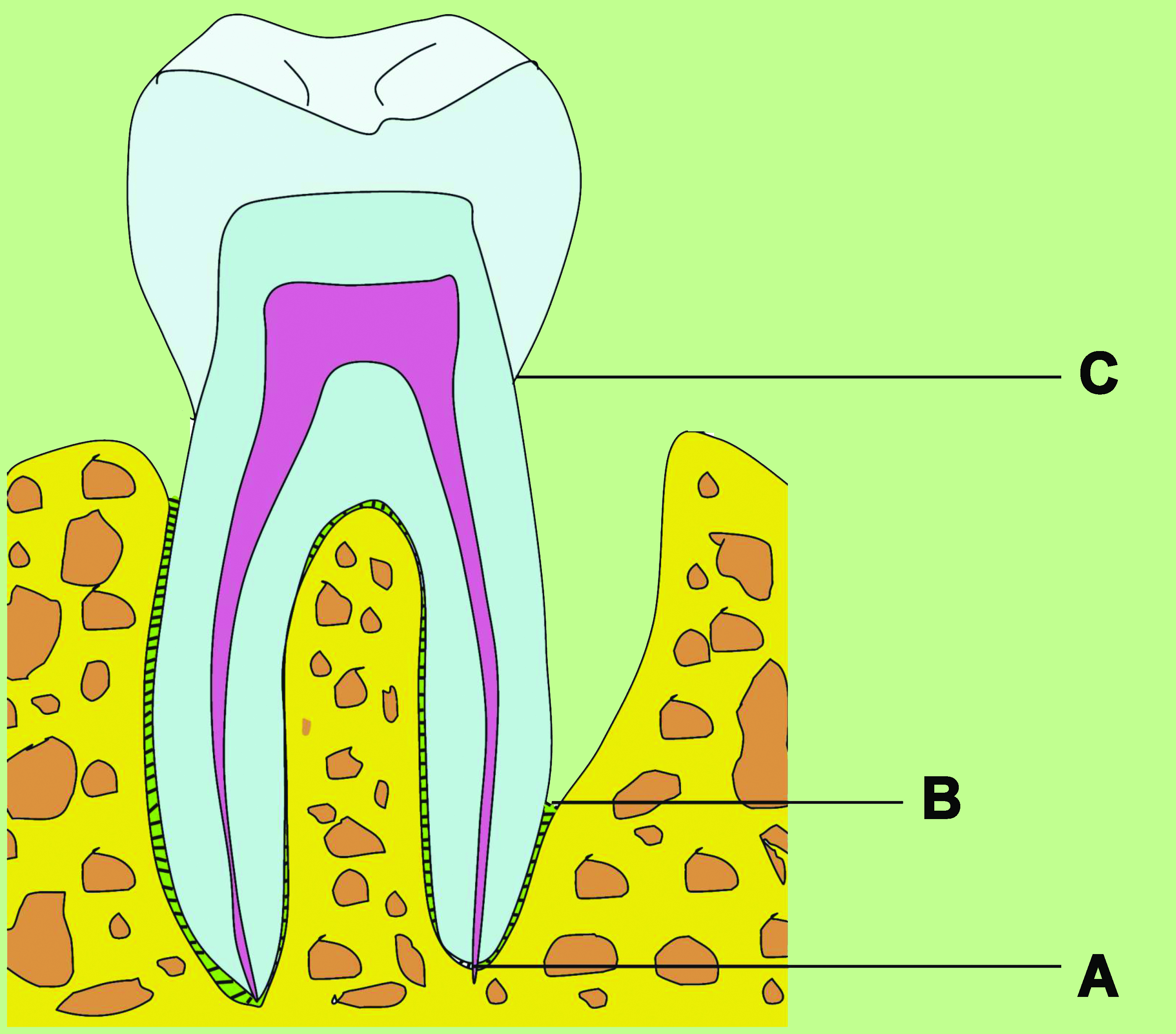


**Figure 2**. Schematic illustration of the bone regeneration assessment using standardized radiography. Points C, A and B represent the cementoenamel junction, apex and bottom of the bone defect, respectively. The examiner measured the tooth axis heights between Points C and A and between Points C and B (bone-defect depth, BDD) on the X-ray for each patient. To adjust for slight errors due to imaging, the measurements from 5 examiners were multiplied by the A-C ratio of the before to after administration to correct for C-B after administration. Based on C-B data before surgery and during follow-up (3, 6 and 12 months), increse of BDD was calculated to serve as an indicator of increase in alveolar bone height post-operation [37,38].

***Inspection of periodontal tissue around the teeth under investigation***

We will measure the items shown below at 6 positions (mesiobuccal, buccal, distobuccal, mediolingual, lingual, and distolingual) around each tooth under investigation.

- Clinical attachment level (CAL): A stent will be prepared for each subject. Using the cementoenamel junction or the margin of the restorative material as the control point, the distance between the control point and bottom of the gingival sulcus will be measured for each test subject using the same periodontal probe.
- Probing depth (PD): Simultaneously with the CAL measurement, we will measure the distance from the gingival margin to the bottom of the gingival sulcus for each subject using the same periodontal probe
- Bleeding on probing (BOP; + or -): The presence of bleeding will be checked 10 s after probing.
- Gingival index (GI): The GI will be determined as described by Löe and Silness [36]
- Mobility of tooth (MO): MO will be determined as described by Miller [37].
- Recession of gingiva (REC): Using the cementoenamel junction or the margin of the restorative material as the control point, the distance between the control point and gingival margin was measured for each subject using the same periodontal probe
- Plaque index (PlI): The PlI will be determined as described by Silness and Löe [38].
- Width of keratinized gingiva (KG): The shortest distance between the coronal gingival margin and the mucogingival junction will be measured for each subject using the same periodontal probe.

**Statistics**

This study will be performed using a per-protocol analysis. In this analysis, all of the randomized teeth will receive at least one therapy, but teeth that do not receive treatment will be excluded (modified per-protocol analysis). The last-observation-carried-forward method will be used for the per-protocol analysis. The missing data points will be input into the post-baseline follow-up visits from the last observation available for each patient. For analysis, we will use SAS version 8.2 software (SAS Institute Inc., Cary, North Carolina, USA). The per-protocol set analysis will be performed for the primary outcome. The baseline between-group comparisons within age and clinical examination indices will be performed using independent group *t* tests. The between-group comparison of sex will be performed using Fisher’s exact probability test. The changes in clinical examination indices will be tested using a repeated-measures analysis of variance. The level of statistical significance will be set at *p*<0.05.

**Ethics and research integrity**

***Fundamental principle***

We will perform this trial in accordance with the Randomized Controlled Principle of trial design.

***Institutional Review Board (IRB)***

Prior to this clinical trial, the study will be reviewed by the IRB at Fourth Military Medical University School of Stomatology for a written approval.

***Ensuring human-rights***

We will comply with the Declaration of Helsinki and place maximum priority on ensuring human rights, welfare and the safety of the subjects. The protocol of this trial will be externally reviewed by anonymous independent ethical review committees to ensure no serious ethical concerns.

**Discussion**

Periodontitis is a chronic, infectious disease of the human periodontium that is characterized by the loss of supporting tissues surrounding the tooth, such as the PDL, cementum and alveolar bone. A major goal of periodontal therapy is the regeneration of the entire supporting structures of teeth. Although a number of treatment modalities are currently available, such as conventional treatments that mechanically remove the bacterial biofilm and the use of platelet-rich plasma, enamel matrix derivatives, and/or recombinant human growth factors that actively induce periodontal regeneration, clinicians continue to seek more predictable regenerative therapies that are less technique-sensitive, lead to fast tissue regeneration, and are applicable to the broad array of periodontal conditions encountered daily in the clinic. Recent evidence from animal models [20-29] and several small-scale pilot/feasibility studies [30-33] indicates that *ex vivo* cultured PDL cells may serve as powerful “tools” for periodontal therapy. Numerous animal studies have provided an overwhelming body of evidence that suggests that MSCs can be used for periodontal regeneration. As a consequence of these successful animal studies, the clinical application of stem cells for the regeneration of periodontal tissue has begun [16].

Thus, these data support the move from animal studies to human clinical trials. However, there are critical steps in moving the field towards human clinical utility. First and foremost, the safety and efficacy of these cell-based therapies has not been fully evaluated, and the risks of stem cell therapies have been underscored by several clinicians and researchers. Second, issues, such as cell delivery, cell immunogenicity, use of autologous cells or allogeneic cells, control of cell fates *in vitro* and *in vivo*, and cost-effectiveness are all important considerations that need to be addressed before this therapy can progress [39,40]. The next critical phase requires the identification of which tissues provide the most appropriate donor source and the systematic validation of specific MSCs that are reliable sources for periodontal cytotherapeutic use. Furthermore, the establishment of large-scale preparation facilities incorporating the stringent protocols of good manufacturing procedures will be an absolute necessity. Finally, regulatory agencies must define new criteria to evaluate the risk associated with specific stem cells and their differentiated progeny (reviewed in [39,40]). The purpose of this trial is to provide evidence to support the use of *ex vivo*-cultured cells to treat periodontitis and determine the best approach for treating this disease.

In the case of incurable and life-threatening diseases, such as diabetes, Parkinson’s, muscular dystrophy, Alzheimer’s, neural and cardiac diseases, and refractory systemic lupus erythematosus, cell-based therapy is more likely to be warranted and accepted by the government and patients [41,42]. However, periodontal tissue regeneration using cell therapy may not be economically viable or competitive with the currently available root canal therapies and dental implants. Due to the non-life-threatening nature of periodontitis, periodontal tissues are not a major target for stem cell-based regenerative medical research. Nevertheless, affected teeth are ideal for the evaluation of new therapies because the patients are not usually ill. Thus, if anything goes wrong with the treatment, the situation is far less likely to be life threatening. Furthermore, the accessibility of teeth facilitates treatments that do not require major surgery.

We expect to complete patient recruitment by December 2014 and anticipate that the results of this study will have implications for oral health care and facilitate improvements in the treatment of people with periodontitis. The results of this study will also allow us to determine if cell-based intervention is safe and effective for human dental use in clinical trials. These results may have important implications for the design of subsequent clinical trials and the development of sustainable, cost-effective cell-products for people with periodontitis.

This study will use central randomization, which is a strict and complete randomization method that ensures adequate concealment. The surgeon and the investigator who collect baseline and follow-up data will work independently in this trial. Throughout the entire trial, the patients are not aware of their group assignment. They will be told only that they will receive a periodontal surgery treatment that potentially includes cell products. This trial is a single-center randomized controlled study of approximately 30 patients that includes a 12-month follow up. This trial will be the first randomized controlled trial to address the effectiveness of cell therapy in combination with GTR and bone replacement for periodontitis.

In conclusion, stem cell therapy is a promising nascent therapy that may lead to the regeneration of lost periodontal tissue. Regenerative dentistry is at the forefront of the transition of basic science research to the clinical reconstructive arena. Although there are many issues that need to be resolved before stem cell therapies become commonplace, clinicians should continue to monitor the progression of these technologies.

**Acknowledgements and funding**

This project will be supported by a translational research grant from the Fourth Military Medical University School of Stomatology. The funding bodies play no role in the study design or the decision to submit the manuscript for publication. The authors acknowledge our previous basic and animal studies in this field, which were supported by grants from the National Natural Science Foundation of China (81471791, 81500853 and 81530050).

**References**

1. Burt B. Position paper: epidemiology of periodontal diseases. J Periodontol 2005: 76: 1406-19.
2. Pihlstrom BL, Michalowicz BS, Johnson NW. Periodontal diseases. Lancet 2005;366:1809-20.
3. Williams RC, Barnett AH, Claffey N, Davis M, Gadsby R, Kellett M, Lip GY, Thackray S. The potential impact of periodontal disease on general health: a consensus view. Curr Med Res Opin 2008: 24: 1635-43.
4. Villar CC, Cochran DL. Regeneration of periodontal tissues: guided tissue regeneration. Dent Clin North Am 2010;54:73-92.
5. Reynolds MA, Aichelmann-Reidy ME, Branch-Mays GL. Regeneration of periodontal tissue: bone replacement grafts. Dent Clin North Am 2010;54:55-71.
6. Lee J, Stavropoulos A, Susin C, Wikesjö UM. Periodontal regeneration: focus on growth and differentiation factors. Dent Clin North Am 2010;54:93-111.
7. Elangovan S, Srinivasan S, Ayilavarapu S. Novel regenerative strategies to enhance periodontal therapy outcome. Expert Opin Biol Ther 2009;9:399-410.
8. Chen FM, Jin Y. Periodontal tissue engineering and regeneration: current approaches and expanding opportunities. Tissue Eng Part B Rev 2010; 16: 219-55.
9. Trombelli L. Which reconstructive procedures are effective for treating the periodontal intraosseous defect? Periodontol 2000 2005;37:88-105.
10. Cortellini P, Labriola A, Tonetti MS. Regenerative periodontal therapy in intrabony defects: state of the art. Minerva Stomatol 2007;56:519-39.
11. Bosshardt DD, Sculean A. Does periodontal tissue regeneration really work? Periodontol 2000 2009;51:208-19.
12. Chapple IL. Periodontal diagnosis and treatment--where does the future lie? Periodontol 2000 2009; 51: 9-24.
13. Seo BM, Miura M, Gronthos S, Bartold PM, Batouli S, Brahim J, Young M, Robey PG, Wang CY, Shi S. Investigation of multipotent postnatal stem cells from human periodontal ligament. Lancet 2004; 364: 149-155.
14. Chen FM, Zhang J, Zhang M, An Y, Chen F, Wu ZF. A review on endogenous regenerative technology in periodontal regenerative medicine. Biomaterials 2010; 31: 7892-927.
15. Bartold PM, McCulloch CA, Narayanan AS, Pitaru S. Tissue engineering: a new paradigm for periodontal regeneration based on molecular and cell biology. Periodontol 2000 24:253-69.
16. Chen FM, Sun HH, Lu H, Yu Q. Stem cell-delivery therapeutics for periodontal tissue regeneration. Biomaterials 2012;33:6320-44.
17. Lin NH, Gronthos S, Mark BP. Stem cells and future periodontal regeneration. Periodontology 2000 2009;51:239-251.
18. Intini G. Future approaches in periodontal regeneration: gene therapy, stem cells, and RNA interference. Dent Clin North Am 2010;54:141-55.
19. Catón J, Bostanci N, Remboutsika E, De Bari C, Mitsiadis TA. Future dentistry: cell therapy meets tooth and periodontal repair and regeneration. J Cell Mol Med 2011;15:1054-65.
20. Dogan A, Ozdemir A, Kubar A, Oygür T. Assessment of periodontal healing by seeding of fibroblast-like cells derived from regenerated periodontal ligament in artificial furcation defects in a dog: a pilot study. Tissue Eng 2002;8:273-82.
21. Doğan A, Ozdemir A, Kubar A, Oygür T. Healing of artificial fenestration defects by seeding of fibroblast-like cells derived from regenerated periodontal ligament in a dog: a preliminary study. Tissue Eng 2003;9:1189-96.
22. Nakahara T, Nakamura T, Kobayashi E, Kuremoto K, Matsuno T, Tabata Y, Eto K, Shimizu Y. In situ tissue engineering of periodontal tissues by seeding with periodontal ligament-derived cells. Tissue Eng 2004;10:537-44.
23. Bruckmann C, Walboomers XF, Matsuzaka K, Jansen JA. Periodontal ligament and gingival fibroblast adhesion to dentin-like textured surfaces. Biomaterials 2005;26:339-46.
24. Yang ZH, Zhang XJ, Dang NN, Ma ZF, Xu L, Wu JJ, Sun YJ, Duan YZ, Lin Z, Jin Y. Apical tooth germ cell-conditioned medium enhances the differentiation of periodontal ligament stem cells into cementum/periodontal ligament-like tissues. J Periodontal Res 2009;44:199-210.
25. Washio K, Iwata T, Mizutani M, Ando T, Yamato M, Okano T, Ishikawa I. Assessment of cell sheets derived from human periodontal ligament cells: a pre-clinical study. Cell Tissue Res 2010;341:397-404.
26. Ding G, Liu Y, Wang W, Wei F, Liu D, Fan Z, An Y, Zhang C, Wang S. Allogeneic periodontal ligament stem cell therapy for periodontitis in swine. Stem Cells 2010;28:1829-38.
27. Yang Y, Rossi FM, Putnins EE. Periodontal regeneration using engineered bone marrow mesenchymal stromal cells. Biomaterials 2010;31:8574-82.
28. Park CH, Rios HF, Jin Q, Bland ME, Flanagan CL, Hollister SJ, Giannobile WV. Biomimetic hybrid scaffolds for engineering human tooth-ligament interfaces. Biomaterials 2010;31:5945-52.
29. Tsumanuma Y, Iwata T, Washio K, Yoshida T, Yamada A, Takagi R, Ohno T, Lin K, Yamato M, Ishikawa I, Okano T, Izumi Y. Comparison of different tissue-derived stem cell sheets for periodontal regeneration in a canine 1-wall defect model. Biomaterials 2011;32:5819-25.
30. Feng F, Akiyama K, Liu Y, Yamaza T, Wang TM, Chen JH, Wang BB, Huang GT, Wang S, Shi S. Utility of PDL progenitors for in vivo tissue regeneration: a report of 3 cases. Oral Dis 2010;16:20-8.
31. Yamada Y, Ueda M, Hibi H, Baba S. A novel approach to periodontal tissue regeneration with mesenchymal stem cells and platelet-rich plasma using tissue engineering technology: A clinical case report. Int J Periodontics Restorative Dent 2006;26:363-9.
32. d'Aquino R, De Rosa A, Lanza V, Tirino V, Laino L, Graziano A, Desiderio V, Laino G, Papaccio G. Human mandible bone defect repair by the grafting of dental pulp stem/progenitor cells and collagen sponge biocomplexes. Eur Cell Mater 2009;18:75-83.
33. McAllister BS. Stem cell-containing allograft matrix enhances periodontal regeneration: case presentations. Int J Periodontics Restorative Dent 2011;31:149-55.
34. Kitamura M, Nakashima K, Kowashi Y, Fujii T, Shimauchi H, Sasano T, Furuuchi T, Fukuda M, Noguchi T, Shibutani T, Iwayama Y, Takashiba S, Kurihara H, Ninomiya M, Kido J, Nagata T, Hamachi T, Maeda K, Hara Y, Izumi Y, Hirofuji T, Imai E, Omae M, Watanuki M, Murakami S. Periodontal tissue regeneration using fibroblast growth factor-2: randomized controlled phase II clinical trial. PLoS One 2008;3:e2611.
35. Kitamura M, Akamatsu M, Machigashira M, Hara Y, Sakagami R, Hirofuji T, Hamachi T, Maeda K, Yokota M, Kido J, Nagata T, Kurihara H, Takashiba S, Sibutani T, Fukuda M, Noguchi T, Yamazaki K, Yoshie H, Ioroi K, Arai T, Nakagawa T, Ito K, Oda S, Izumi Y, Ogata Y, Yamada S, Shimauchi H, Kunimatsu K, Kawanami M, Fujii T, Furuichi Y, Furuuchi T, Sasano T, Imai E, Omae M, Yamada S, Watanuki M, Murakami S. FGF-2 stimulates periodontal regeneration: results of a multi-center randomized clinical trial. J Dent Res 2011;90:35-40.
36. Loë H, Silness J. Periodontal disease in pregnancy. I. Prevalence and severity. Acta Odontol Scand 1963;21: 533-51.
37. Miller SC. Textbook of Periodontia, 1950, 3rd ed. Philadelphia and Toronto: The Blakiston Co.
38. Silness J, Loë H. Periodontal disease in pregnancy. II. Correlation between oral hygiene and periodontal condition. Acta Odontol Scand 1964;22:121-35.
39. Hynes K, Menicanin D, Gronthos S, Bartold PM. Clinical utility of stem cells for periodontal regeneration. Periodontol 2000 2012;59:203-27.
40. Yoshida T, Washio K, Iwata T, Okano T, Ishikawa I. Current status and future development of cell transplantation therapy for periodontal tissue regeneration. Int J Dent 2012;2012:307024.
41. Daley GQ, Scadden DT. Prospects for stem cell-based therapy. Cell 2008; 132: 544-8.
42. Lalu MM, McIntyre L, Pugliese C, Fergusson D, Winston BW, Marshall JC, Granton J, Stewart DJ; Canadian Critical Care Trials Group. Safety of Cell Therapy with Mesenchymal Stromal Cells (SafeCell): A Systematic Review and Meta-Analysis of Clinical Trials. PLoS One 2012;7:e47559.
