# Supplementary material for: Treatment of periodontal intrabony defects using autologous periodontal ligament stem cells: a randomized clinical trial
Source: Stem Cell Res Ther. 2016 Feb 19;7:33. doi: 10.1186/s13287-016-0288-1 (PMC4761216; doi:10.1186/s13287-016-0288-1)
Supplement: Additional file 2: — Appendices (inclusion and exclusion criteria of the trial; detailed methods for randomization; detailed methods for cell isolation, characterization and cell transplant preparation; and detailed methods for determining bone fill). (DOCX 2833 kb) [file 13287_2016_288_MOESM2_ESM.docx]

***Additional file 2***

**Appendices**

**Treatment of periodontal intrabony defects using autologous periodontal ligament stem cells: A randomized clinical trial**

**Fa-Ming Chen^1,2^, Li-Na Gao^1,2^, Bei-Min Tian^1,2^, Xi-Yu Zhang^1,2^, Yong-Jie Zhang ^2,3^, Guang-Ying Dong^1,2^, Hong Lu^1,2^, Qing Chu^1,2^, Jie Xu^1,2^, Yang Yu^1,2^, Rui-Xin Wu^1,2^, Yuan Yin^1,2^, Songtao Shi ^2,4^, Yan Jin ^2,3^**

^1^ State Key Laboratory of Military Stomatology, Department of Periodontology, School of Stomatology, Fourth Military Medical University, Xi’an, Shaanxi, P. R. China

^2^ Shaanxi Key Laboratory of Stomatology, Translational Research Team, School of Stomatology, Fourth Military Medical University, Xi’an, Shaanxi, P. R. China

**Appendix 1. Inclusion and exclusion criteria** [1,2]

***Inclusion criteria***

- those ≥18 years old and <65 years old;
- those diagnosed with a 2- or 3-walled vertical intrabony defect ≥3 mm deep from the top of the remaining alveolar bone from radiography and clinical periodontal parameters;
- those who have accomplished the initial preparation and shown good compliance;
- those with a mobility of Degree 2 or less for the tooth under investigation [3] and with a width of the attached gingiva for which the existing GTR and bone graft implantation treatments are considered appropriate;
- those for whom supportive periodontal treatment is applicable (the tooth has the potential to be maintained for at least 3 years) and in accordance with the usual post-operative procedures following flap operation and GTR treatment;
- those whose oral hygiene is well established and who are able to perform appropriate tooth brushing and cleaning following instructions from the investigators and/or sub-investigators after investigational drug administration;
- those who have at least one tooth (e.g., wisdom tooth) that needs to be extracted due to impacted or non-functional reasons and who agree to the tooth extraction;
- those who understand the purposes of the trial and are capable of making an independent decision to comply with trial requirements.

***Exclusion criteria***

- those who took a calcium antagonist during the 4 weeks prior to surgery;
- those in need of the administration of adrenal cortical steroids (equivalent to >20 mg/day of Predonin) within 4 weeks after the surgery;
- those scheduled to undergo a surgical operation in the vicinity of the tooth to be investigated within 36 weeks after surgery;
- those with coexisting mental or consciousness disorders [4];
- those with coexisting malignant tumors or history of the same;
- those with coexisting diabetes (HbA_1C_ >6.5%);
- those in extremely poor nutritional conditions (serum albumin concentration <2 g/dL);
- those with ≥200 mL of blood drawn during the 4 weeks prior to surgery;
- those given another investigational drug during the 24 h preceding our investigational drug administration;
- those with a coexisting disorder of the kidney, liver, blood and/or circulatory system (Grade 2 or above);
- those who are either pregnant, possibly pregnant or breast-feeding or who hope to become pregnant during the trial period;
- those with a previous history of hypersensitivity to any biologically active drugs;
- those who smoked more than 10 cigarettes during the past 3 months;
- those who are involved with the research team of this trial;
- those with any other condition, as determined by the investigators or sub-investigators, that could make the subject unsuitable for the trial, impair the validity of the informed consent, or impair the subject's suitability for the trial.

**Appendix 2. Methods for randomization**

Each patient was examined by at least five independent principal investigators. Investigator 1 performed a basic oral examination using a clinical inspecting apparatus and X-ray, checked all inclusion and exclusion criteria and ensured the informed consents were signed by the patients at the Registration Center. Next, Investigator 2 performed the pre-surgical treatments, re-checked the inclusion and exclusion criteria, and extracted the tooth/teeth that was/were subjected to cell isolation by Investigator 4. Investigator 2 selected no more than 2 teeth (from different regions) to be investigated in each patient. Following the collection of baseline data from the selected tooth/teeth by Investigator 3, Investigator 4 cultured the cells, made the cell products, and created an allocation table consisting of 2 cases per block that were allocated to the Control group (Bio-oss® only) and Cell group (Bio-oss® and cell sheets). According to this allocation table, a label indicating the corresponding group was attached to each tooth; thus, each tooth was randomly assigned to one of the two treatment groups. The maximum number of teeth involved from one patient was 2; hence, if one tooth was allocated to the Control group, the other was automatically assigned to the Cell testing group. However, if a patient only had one tooth chosen for investigation, it had a fair chance to be allocated to either group. Once a tooth was assigned to a group, it was kept in a secure locked drawer inaccessible to all other study personnel. All of the patients received oral hygiene instructions, a tooth cleaning and basic dental therapies if needed, such as cavity filling and occlusal adjustment. The periodontal surgery was performed 4 to 5 weeks after these pre-surgical treatments. When the oral condition and cell products were both ready for surgery, Investigator 4 was asked to give the assigned implants (Bio-oss® only or a combination of Bio-oss® and cell sheets) to the physician (Investigator 5), who then performed the surgery. This procedure ensured that the randomization was not influenced by the physicians taking part in this study. Investigator 4 recorded the patients’ details and the number of the envelope that was assigned to each tooth. Investigator 3 performed the follow-up study (data collection, safety assessment, etc.) of the patient, and the blind was not broken until the clinical trial was completed.

**Appendix 3. Methods for cell isolation, characterization and cell transplant preparation**

The third molars of patients in the Cell group were extracted and subjected to cell isolation and transplant production according to Good Laboratory Practice and Good Manufacturing Practice guidelines. Prior to extraction surgery, at least 2 independent assessors must have concluded that a tooth or teeth needed extraction due to impaction or non-functional reasons.

***Cell isolation***

Cells were isolated from the patient’s own PDL tissues of the third molar. Briefly, extracted teeth were rinsed with phosphate-buffered saline (PBS; Gibco, Grand Island, NY, USA) for 3 min for a total of 5 times. The PDL was gently separated from the surface of the root and digested in a solution of 0.2% collagenase type I (Sigma) for 15 min at 37°C. Single-cell suspensions were obtained by passing the solution through an 80-mesh strainer and centrifuging at 1000 r/min for 8 minutes. Cultures were grown in α-minimum essential medium (α-MEM, Invitrogen, Carlsbad, CA) containing 100 units/mL penicillin and 100 mg/mL streptomycin (Invitrogen) supplemented with 10% fetal bovine serum (FBS). After cell counting, the cultures were inoculated in 25-cm^2^ culture flasks with 4 mL of α-MEM medium at a concentration of 5×10^5^ per flask and incubated in 5% CO_2_/95% air at 37°C. The culture medium was changed once every two days until the cells grew to 80% confluence (7-10 days), at which time the cells were passaged.

***Passage of cells***

After washing away the remaining culture medium in the culture flask using PBS, the cells were digested with 0.25% trypsin for 2 min at 37°C. α-MEM supplemented with 10% fetal bovine serum was added to stop the digestion until the cells observed under a reverse microscope appeared to be circular in shape and floating. Next, the cells were centrifuged at 800-1000 r/min for 6 minutes and then re-suspended in PBS. A unicellular suspension was inoculated into the culture flask at a concentration of 5×10^5^/mL. The cell type, passage, cell density, date and operator were recorded. The number of cells at passage 4 was 1×10^7^ (passage time: approximately 20 days). The obtained cells were identified by adipogenic differentiation and osteogenic differentiation.

***Cell characterization***

To analyze the surface antigens, PDLSCs were washed in phosphate buffered saline (PBS) and then incubated with 2 μg/mL of the following mouse anti-human monoclonal antibodies for 30 min at 4 °C: CD29, CD44, CD90, CD105, CD34, CD45, STRO-1 and CD146. To induce osteoblastic differentiation, 10^5^ PDLSCs were seeded in 6-well plates and cultured. When cells reached subconfluence, the culture medium was changed to α-MEM containing ascorbic acid (L-ascorbic acid 2-phosphate) (50 μg/mL) (Sigma–Aldrich), dexamethasone (10^−8^ M) (Sigma–Aldrich) and β-glycerophosphate (10 mM) (Sigma–Aldrich). The culture medium was changed every 3 days for either 14 or 28 days. For Alizarin Red staining, cells were fixed with 70% ethanol for 15 min at room temperature and stained using a 1% Alizarin Red solution for 5 min at room temperature. After staining, the culture plate was washed with tap water and air-dried.

To induce adipocyte differentiation, 10^5^ PDLSCs were seeded in 6-well plates and cultured in growth medium. Adipocyte differentiation was induced using adipocyte differentiation medium containing 1 μmol/L dexamethasone, 0.5 mmol/L IBMX, 10 μg/mL insulin, and 100 mmol/L Indomethacin until day 14. The cells were then fixed using 70% ethanol for 15 min and washed twice with PBS. Oil Red O solution (Sigma) was added for 15 min, followed by rinsing of the plate with PBS.

***Cell transplants***

To create the cell sheet, the PDLSCs were digested by trypsin to obtain single cell suspensions and then inoculated on 6-well plates at 1×10^5^ per well with L-ascorbic acid (vitamin C; 30 μg/mL, Sigma) until confluent (approximately day 10). The cells were then assessed for their morphology and sheet-forming capacity. After a 10-day culture, white membranous substances appeared on the bottom of the wells. The cells within the sheets (underwent approximately 30-day *ex-vivo* culture) were collected to evaluate their chromosomal stability compared to freshly isolated PDLSCs. Briefly, 1×10^6^ cells were incubated with 0.1 μg/mL colchicine for 3 h and treated with 0.075 mol/L potassium chloride solution for another 30 min at 37°C. After centrifugation, cell deposits were resuspended in the mixed stationary liquid (consisting of 75% methanol and 25% glacial acetic acid) and then incubated in a water bath (37°C) for 30 min. Next, 1 drop of the cell suspension was added to the slide center and pretreated with 0.25% trypsin for 2 min. Finally, chromosomes of PDLSCs were dyed using a 10% Giemsa stain and photographed under an inverted microscope. No chromosomal karyotype changes for PDLSCs occurred during a 30-day *ex-vivo* culture involving expansion and cell sheet induction (Supp. Fig. 1). Before clinical application, the culture medium was discarded, and the cell sheets were rinsed twice with PBS. Next, 0.25 g of Bio-Oss® particulates were distributed on the surface of the cell sheet (i.e., a concentration of 0.25 g of materials per sheet). The PDLSC cell sheets were rolled up to pack the Bio-Oss® particulates, which were freshly prepared for clinical treatment. Upon implantation, the level at which Bio-Oss® particulates was placed was 1 mm over the crestal height of the defect. Depending on the size of the defects, the number of cells in each defect varied. However, the cell number per unit of defect volume was the same.


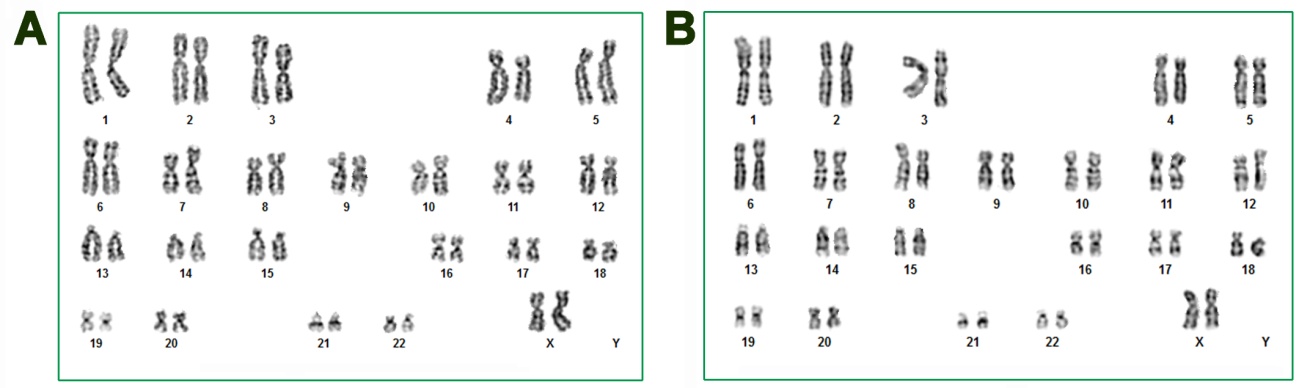


**Supp. Fig. 1**. The chromosomal stability of PDLSCs was assessed using chromosomal karyotype changes during an *ex-vivo* culture involving cell expansion, cell passage and cell sheet induction. There were no karyotype alterations after a 30-day *ex-vivo* culture of patient-matched PDLSCs (**B**) when compared with the freshly isolated cells (**A**).

**Appendix 4. Methods for determining bone fill (shown as a decrease in the bone-defect depth), CAL, PD and GR**

***Standardized radiography for regions of investigation*** [1,2]

Our geometrically standardized radiography employed dental film (Kodak InSight Super Poly-Soft; Eastman Kodak Company, New York, USA) and photograph indicators (Cone Indicator-II; Hanshin Technical Laboratory, Hyogo, Japan) that were customized with resin stents. Two doctors specializing in dental radiology from the Department of Oral Diagnosis at Fourth Military Medical University School of Stomatology measured the distance (millimeters, mm) from the deepest part of the defect to the cementoenamel junction of the tooth using the methods described in the literature (Supp. Fig. 2) [5]. Each examiner independently measured this rate while blinded to the group assignments. The median of 2 measurements taken from the same image was selected for the efficacy analysis.


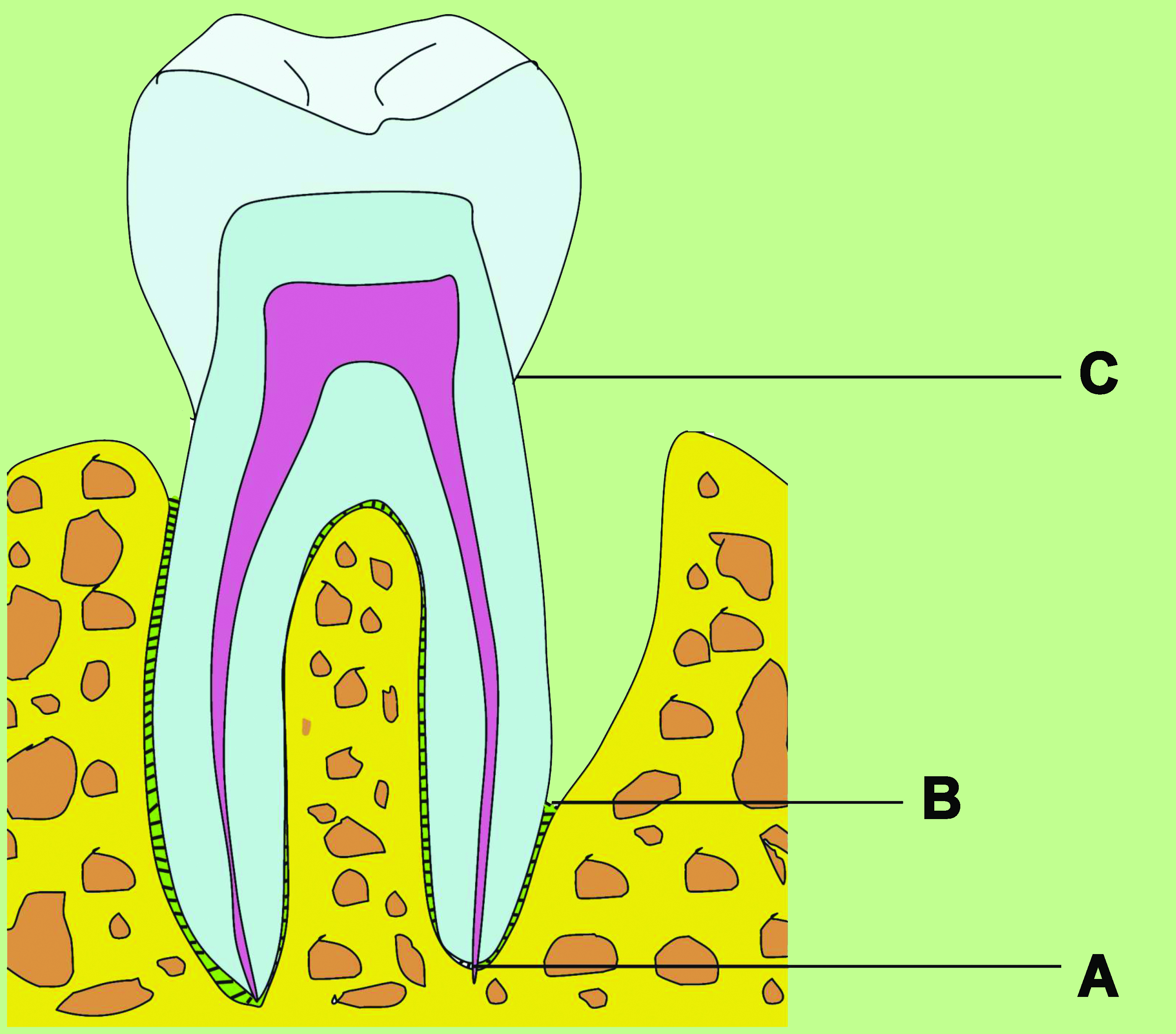


**Supp. Fig. 2**. Schematic illustration of the bone regeneration assessment using standardized radiography. Points C, A and B represent the cementoenamel junction, apex and bottom of the bone defect, respectively. The examiner measured the tooth axis heights between Points C and A and between Points C and B (bone-defect depth, BDD) on the X-ray for each patient. To adjust for slight errors due to imaging, the measurements from 5 examiners were multiplied by the A-C ratio of the before to after administration to correct for C-B after administration. Based on C-B data before surgery and during follow-up (3, 6 and 12 months), increse of BDD was calculated to serve as an indicator of increase in alveolar bone height post-operation [6,7].

***Inspection of periodontal tissue around the tooth under investigation***

We performed the measurements listed below at 6 positions (mesiobuccal, buccal, distobuccal, mediolingual, lingual, and distolingual) around each tooth under investigation.

- Clinical attachment level (CAL): A stent was prepared for each subject. Using the cementoenamel junction or the margin of the restorative material as the control point, the distance between the control point and bottom of the gingival sulcus was measured for each test subject using the same periodontal probe.
- Probing depth (PD): Simultaneously with the CAL measurement, the distance from the gingival margin to the bottom of the gingival sulcus was measured for each subject using the same periodontal probe
- Recession of gingiva (GR): Using the cementoenamel junction or the margin of the restorative material as the control point, the distance between the control point and gingival margin was measured for each subject using the same periodontal probe.

**Appendix 5. References for Additional file 2**

1. Kitamura M, Nakashima K, Kowashi Y, Fujii T, Shimauchi H, Sasano T, Furuuchi T, Fukuda M, Noguchi T, Shibutani T, Iwayama Y, Takashiba S, Kurihara H, Ninomiya M, Kido J, Nagata T, Hamachi T, Maeda K, Hara Y, Izumi Y, Hirofuji T, Imai E, Omae M, Watanuki M, Murakami S: **Periodontal tissue regeneration using fibroblast growth factor-2: randomized controlled phase II clinical trial**. *PLoS One* 2008, **3**:e2611.
2. Kitamura M, Akamatsu M, Machigashira M, Hara Y, Sakagami R, Hirofuji T, Hamachi T, Maeda K, Yokota M, Kido J, Nagata T, Kurihara H, Takashiba S, Sibutani T, Fukuda M, Noguchi T, Yamazaki K, Yoshie H, Ioroi K, Arai T, Nakagawa T, Ito K, Oda S, Izumi Y, Ogata Y, Yamada S, Shimauchi H, Kunimatsu K, Kawanami M, Fujii T, Furuichi Y, Furuuchi T, Sasano T, Imai E, Omae M, Yamada S, Watanuki M, Murakami S.: **FGF-2 stimulates periodontal regeneration: results of a multi-center randomized clinical trial**. *J Dent Res* 2011, **90**:35-40.
3. Laster L, Laudenbach KW, Stoller NH: **An evaluation of clinical tooth mobility measurements**. J Periodontol. 1975, **46**:603-7.
4. Pagey B, Deering D, Sellman D: **Retention of adolescents with substance dependence and coexisting mental health disorders in outpatient alcohol and drug group therapy**. Int J Ment Health Nurs 2010, **19**:437-44.
5. de Molon RS, Morais-Camillo JA, Sakakura CE, Ferreira MG, Loffredo LC, Scaf G: **Measurements of simulated periodontal bone defects in inverted digital image and film-based radiograph: an in vitro study**. *Imaging Sci Dent.*2012, **42**:243-247.
6. Loë H, Silness J: **Periodontal disease in pregnancy. I. Prevalence and severity**. Acta Odontol Scand 1963, **21**:533-51.
7. Miller SC: **Textbook of Periodontia**, 3rd ed. Philadelphia and Toronto: The Blakiston Co. 1950.
